# Fast electronic resistance switching involving hidden charge density wave states

**DOI:** 10.1038/ncomms11442

**Published:** 2016-05-16

**Authors:** I. Vaskivskyi, I. A. Mihailovic, S. Brazovskii, J. Gospodaric, T. Mertelj, D. Svetin, P. Sutar, D. Mihailovic

**Affiliations:** 1 Complex matter F7, Jozef Stefan Institute, Jamova 39, SI-1000 Ljubljana, Slovenia; 2 Faculty of Mathematics and Physics, University of Ljubljana, Jadranska 19, SI-1000 Ljubljana, Slovenia; 3 Faculty of Electrical Engineering, University of Ljubljana, Tržaška 21, SI-1000 Ljubljana, Slovenia; 4 LPTMS-CNRS, UMR8626, Université Paris-Sud, F-91405 Orsay, France; 5 National University of Science and Technology MISiS, Leninski av. 4, Moscow 119049, Russia; 6 Jozef Stefan International Postgraduate School, Jamova 39, SI-1000 Ljubljana, Slovenia

## Abstract

The functionality of computer memory elements is currently based on multi-stability, driven either by locally manipulating the density of electrons in transistors or by switching magnetic or ferroelectric order. Another possibility is switching between metallic and insulating phases by the motion of ions, but their speed is limited by slow nucleation and inhomogeneous percolative growth. Here we demonstrate fast resistance switching in a charge density wave system caused by pulsed current injection. As a charge pulse travels through the material, it converts a commensurately ordered polaronic Mott insulating state in 1T–TaS_2_ to a metastable electronic state with textured domain walls, accompanied with a conversion of polarons to band states, and concurrent rapid switching from an insulator to a metal. The large resistance change, high switching speed (30 ps) and ultralow energy per bit opens the way to new concepts in non-volatile memory devices manipulating all-electronic states.

## Abstract (web summary)

The control of a material's state via external stimuli is the basis of modern information storage technology. Here, the authors use pulsed currents to induce fast switching between Mott insulator and metallic states in the charge density wave system 1T-TaS_2_, presenting an all-electronic storage mechanism.

## Graphical abstract

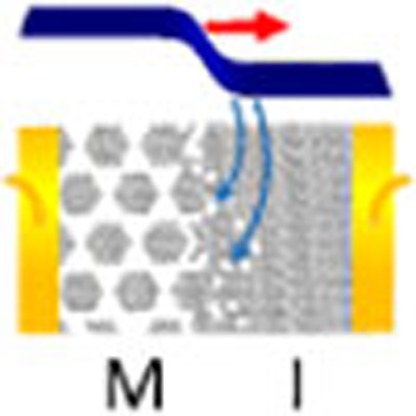

Systems with first-order transitions between competing states in the phase diagram are obvious candidates for resistive memories. In the last few years, pulsed laser experiments have led to an improved understanding of non-equilibrium phase transitions^1^,^2^,^3^,^4^ and metastable hidden (H) states^1^,^2^,^3^,^5^. Optical memory, even though it can be switched with short pulses (35 fs) in charge density wave (CDW) systems^2^ would be severely limited by associated disk-based technology. Electrical switching of CDW systems on the other hand has until now met the fundamental problem that injected charge does not directly couple to the order parameter. Instead, an applied electric field usually causes an incommensurate (IC) CDW to slide^6^, and in experiments so far the system invariably reverts back to the original state when the external field is switched off^7^,^8^.

In this study we describe ultrafast non-volatile resistance switching with short pulsed current injection in 1T–TaS_2_, where injected charges create domain walls (DWs) via coupling to gradients of the CDW order parameter, converting the material from an insulator to a metal at low temperatures. From a technological viewpoint, this opens up the possibility of low-temperature ultrafast memory devices, whose absence has so far seriously impeded progress of ultrafast energy-efficient superconducting computing, for example^9^.

## Results

### Thermal phases of 1T–TaS_2_

**Figure 1:**
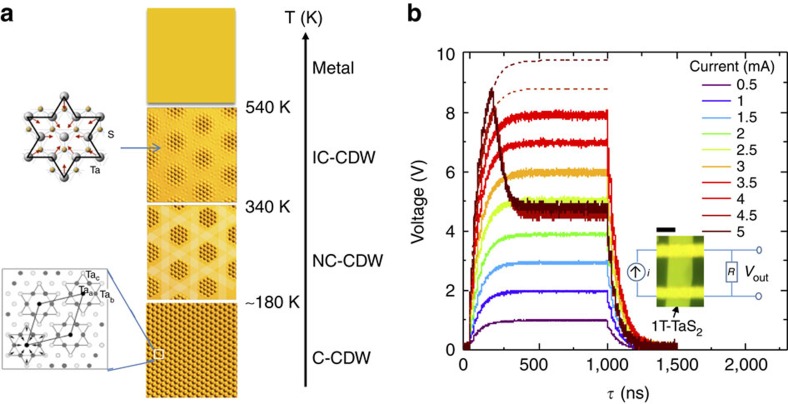
The equilibrium phases of 1T–TaS_2_ and demonstration of switching behaviour. (**a**) An illustration of the different CDW states in 1T–TaS_2_ at different temperatures. (**b**) Temporal behaviour of switching from HI to LO resistance in response to a pulse from a current source. The extrapolated voltage in the absence of switching is shown by the dashed lines. The time–response 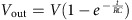 is determined by *τ*_*RC*_=*RC* where *R* is the two-terminal circuit resistance and changes from 53 to 44 ns. Fits of the relaxation times show that the difference between *τ* after switching and *τ*_*RC*_ at the end of the pulse is<0.3 ns, setting a limit on the intrinsic speed of the device. The insert shows the measuring circuit. The small superimposed oscillations are due to ringing of the circuit. Scale bar, 2 μm.

The experiments were performed on 1T–TaS_2_, which at high temperatures is a simple metal with a single Ta *d* electron band crossing the Fermi level. At *T*_c0_=543 K, it forms an IC CDW. On cooling below *T*_c1_=350 K, in trying to conform with the underlying lattice, the IC structure forms a nearly commensurate (NC) state in which electrons on each 13th Ta atom localize, leading to a regular hexagonal array of NC polaron clusters separated by DWs (illustrated in Fig. 1a). Below *T*_c2_=183 K, the DWs vanish and a fully commensurate (C) hexagonal polaronic superlattice forms, which is believed to be a Mott insulator with an energy gap Δ_Mott_≃0.1 eV (refs 10, 11).

### Switching by current pulse

The switching experiments were performed in the Mott state on thin (50–100 nm) single crystal flakes of 1T–TaS_2_ on sapphire substrates, with electrical contacts deposited over the crystal using laser direct lithography, typically 1–8 μm apart. Electrical measurements were made in the C state with pulse lengths between 1 s and 40 ps (see Methods section, Supplementary Note 1 and Supplementary Fig. 1 for details). In Fig. 1b, we show the voltage across the sample at 150 K as a function of time after the application of a 1-μs pulse from a current source for different magnitudes of pulse current. At low pulsed currents, the voltage across the sample follows the source, with rise and fall times defined by the *RC* constant of the circuit *τ*_*RC*_=*R*_HI_*C*=53 ns, where *R*_HI_ is the intial (high) value of circuit resistance. With increasing current, above a certain threshold current, the voltage across the sample drops sharply to ∼60% of its expected value (dashed) and remains constant until the end of the pulse. After switching, *τ*_*RC*_ decreases to 44 ns, consistent with the change of sample resistance (to a value designated as *R*_LO_) after switching. Comparing exponential fits with the fall time *τ*_s_ after switching and the fall time after the end of the pulse we find that they are indistinguishable from each other to within ±0.3 ns, defining an upper limit on the intrinsic switching time. The recovery of *R* to the initial value *R*_HI_ occurs within *τ*_R_∼10 ms, consistent with relaxation time data at 150 K, which changes with temperature *T* as 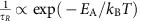, with *E*_A_=280∼2,300 K, depending on the sample substrate^5^, *k*_B_ is the Boltzmann constant.

**Figure 2:**
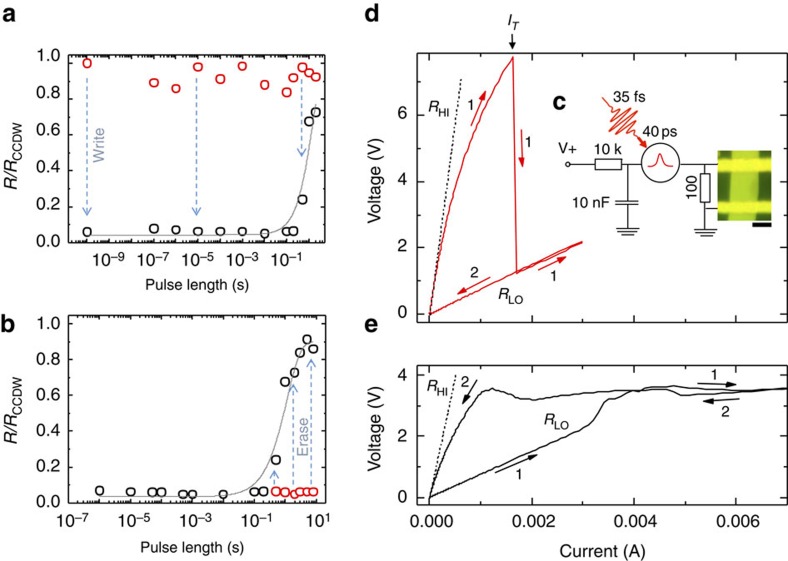
Switching speed and threshold behaviour. (**a**) Switching resistance ratio measured with a single pulse above threshold *I*_T_, each time starting from the HI resistance state (red circles). Black circles show resistivity after current pulse. (**b**) Erasing with 1-s pulses, each time starting from the LO resistance state (red circles). Black circles show resistivity after applying erase pulse. The data were obtained by alternating write/erase cycles and increasing the pulse length. (**c**) The circuit used for the 30-ps measurement with an metal–semiconductor–metal (MSM) device as the current source. (**d**) The voltage *V* measured during each pulse, first incrementally increasing and then decreasing the pulse current *I*, shown by paths (1) and (2), respectively. Above the threshold current *I*_T_, *V* abruptly drops between two consecutive current values. (**e**) Same as in **d**, but starting from the low resistance state increasing *I*_pulse_ along path (1) and then reducing *I*_pulse_ along (2). In all cases *T*=20 K and the pulse length was 10 μs. Scale bar, 2 μm.

To investigate the switching speed, we apply current pulses of different duration and measure the initial and final resistance with a low current after each pulse. For *τ*_pulse_>25 ns, we used a standard electrical pulse generator, whereas for *τ*_pulse_<25 ns we used a 35-fs optical pulse-triggered 30 ps risetime (∼40 ps full width at half maximum) metal–semiconductor–metal pulse source with the 1T–TaS_2_ sample mounted in a 50-Ω transmission line circuit (shown in Fig. 2c). Remarkably, provided that *I*>*I*_T_, we observe switching between *R*_HI_ and *R*_LO_, irrespective of the duration of the applied pulse over the interval 40 ps≲*τ*_pulse_<0.1 s (summarized in Fig. 2a). For *τ*_pulse_>0.1 s, switching is incomplete and does not occur at all for *τ*_pulse_>1 s. Repeating the same experiment, but starting in the low resistance state *R*_LO_, switching from *R*_LO_ to *R*_HI_ occurs for *τ*_pulse_>0.1 s (Fig. 2b). The implication of these measurements is that the *R*_HI_→*R*_LO_ switching is caused by the leading edge of the pulse, in a time *τ*_s_<30 ps. On the other hand, the reverse switching *R*_LO_→*R*_HI_ with *τ*_pulse_⩾0.1 s is consistent with Joule heating above 100 K, where the lifetime is sufficiently short to relax the H state^5^. We label the two cycles described above as write (W) and erase (E).

To systematically measure threshold current *I*_T_, we incrementally increase and then decrease the current pulse magnitude, while simultaneously recording the voltage across the sample. The resulting hysteretic *I–V* curve measured at 14 K for a 80-nm-thick crystal with 8 μm between inner contacts and *τ*_pulse_=50 μs is shown in Fig. 2d. Before switching, the *I–V* curve shows an unusual exponentially increasing *I* with *V*:


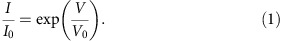


Increasing *I* beyond a critical value *I*_T_, we observe a sharp drop in *V*. Thereafter, the *I–V* curve becomes ohmic over a large range of *I*. On the return cycle (and subsequent cycles as long as *I*<2*I*_T_), the *I–V* curve is linear, with a slope 
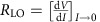
 at the origin that is two to three orders of magnitude smaller than *R*_HI_ in the C state (Fig. 2a). Repeating the measurement cycle, but starting with the sample in the low resistance state *R*_LO_, the response departs from linearity beyond *I*∼*I*_T_/2 (Fig. 2e). Thereafter, *V* increases very slowly with *I*, becoming nearly independent of current above 4 mA. In the return part of the cycle, the nearly *I*-independent behaviour remains until ∼1 mA, whereupon the system reverts to the original C state with *R*_HI_ below 1 mA. More details are presented in Supplementary Fig. 2.

### Temperature dependence of the switching

**Figure 3:**
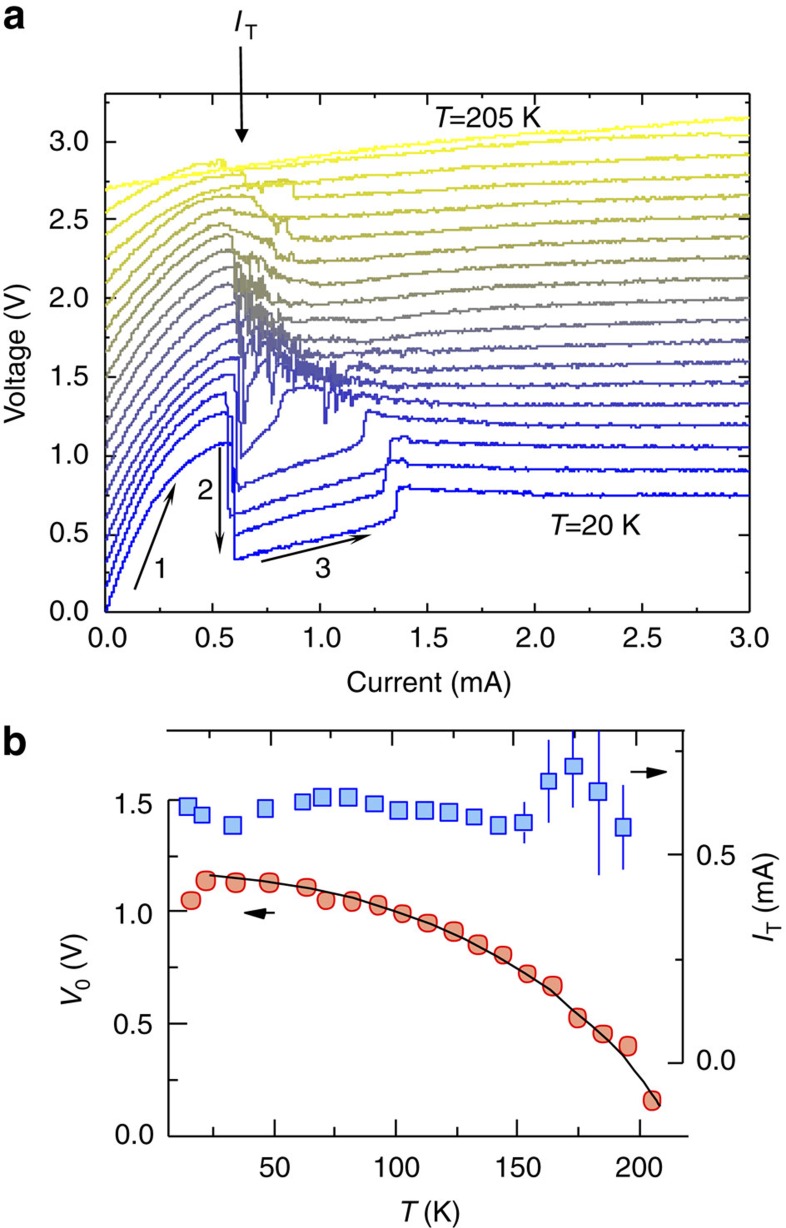
The *T*-dependence of switching. (**a**) The pulsed *V–I* characteristic as in Fig. 2, measured at different temperatures and at 10 K intervals with *τ*_pulse_=50 μs. The *V–I* curve is measured in pulsed mode, by increasing the current incrementally and measuring the voltage at each point. The resistance drops sharply in a few consecutive measurements at the critical value of *I*_T_. The sample was reset in between each *V–I* curve by heating it above 310 K and then cooling down to the indicated temperature for the next measurement. (**b**) *I*_T_ and *V*_0_ as a function of temperature. Error bars are obtained from fitting the data with the exponential function.

The temperature dependence of the switching is shown in Fig. 3a for 20 K<*T*<205 K. For 150 K and below, each curve is measured on a freshly ordered CDW state, prepared by slowly cooling the sample from 150 K to the indicated base temperature before each measurement. Above 150 K, the temperature was gradually increased. For *T* up to ∼55 K, the behaviour is similar to that described above for *T*=14∼20 K (Fig. 2d,e). In the range 55∼165 K, an instability is observed above *I*_T_ , where the voltage fluctuates with each pulse between *R*_HI_ and *R*_LO_. Above 165 K, these fluctuations disappear and the switching ratio *R*_HI_*/R*_LO_ eventually disappears completely above 195 K. The *T*-dependence of *I*_T_ and *V*_0_ obtained from a fit to the data using equation (1) is shown in Fig. 3b. Remarkably, fits to the *I–V* curves below threshold show that *I*_T_ is almost completely independent of *T*, whereas *V*_0_ gradually drops with *T*. (The details of the fitting are given in the Supplementary Note 2 and Supplementary Fig. 3.) The latter behaviour is probably not entirely intrinsic, as at high temperatures the measurement time becomes comparable with the relaxation lifetime *τ*_R_ of the low resistance state^5^. We also note the rather remarkable behaviour in Fig. 3a at high currents, displaying a cross-over from negative differential resistance (DR) through zero DR to a slight positive DR with increasing temperature up to 200 K.

## Discussion

Before discussing the switching mechanism, we first highlight the unusual observations. The switching is remarkably fast and works as a latch with a very sharp current threshold, which cannot be associated with dielectric breakdown into filamentary paths involving ionic motion, as in chalcogenide glasses^7^,^12^, manganite Mott insulators^13^ or band insulator memristors such as TiO_*x*_ (ref. 14). Zener tunnelling and avalanche breakdown can also be eliminated, because they do not exhibit metastability. The pulsed *I–V* curve below switching threshold is also qualitatively different from the observed behaviour for sliding and de-pinning of a CDW^6^,^15^,^16^. On the other hand, well above *I*_T_, the negative DR behaviour gradually crosses over to a small positive DR with increasing temperature, which is reminiscent of dissipationless CDW sliding conductivity (Frölich conductivity) observed in blue bronze at similar current densities^17^.

In previous optical switching experiments, electrons (e) and holes (h) are homogeneously photoexcited and spatially overlapping^2^. The combination of narrow spectral width and unchanged spectral intensity of the collective amplitude mode in the photoexcited H state compared with the C state indicate that the two states are comparably homogeneous. Presently, the e and h are spatially separated at either electrode so the optical mechanism does not apply and we expect the structure to be less homogeneous. Scanning tunneling microscope (STM) images of the sample surface after current injection by an STM tip confirm this: they show an irregular meander of DWs rather than a regular array of DWs (see Supplementary Figs 4 and 5)^18^,^19^. In our experiments we find that different *R*_LO_ with different relaxation properties can be reached with different pulse voltages near threshold (see Supplementary Note 3 for a few examples), indicating that different DW configurations can be created under different conditions. The free energy landscape of H states in 1T–TaS_2_ clearly has multiple minima—consistent with the notion of an electronic glass.

**Figure 4:**
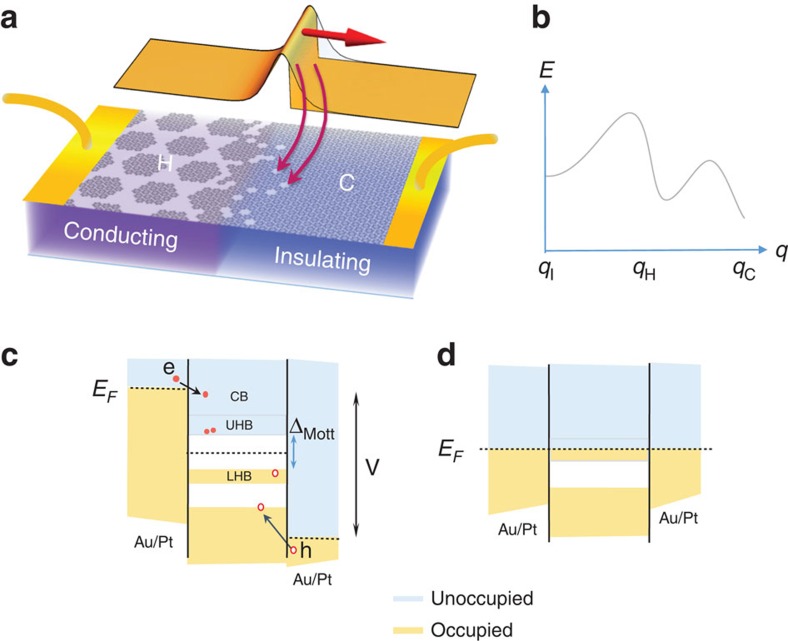
An illustration of carrier trapping and DW formation after carrier injection. (**a**) The injected charges are trapped in the C structure causing the formation of a textured glassy electronic state. The charge density *ρ* is depleted at the leading edge of the pulse as it travels through the sample. (**b**) The free energy as a function of CDW wavevector based on the model of Nakanishi and Shiba^20^. *q*_C_ and *q*_I_ are the *q*-vectors of the undoped commensurate and IC states, respectively. The *q*-vector in the H state *q*_H_ is determined by the density of carriers forming the DWs. (**c**) A depiction of the charge injection at the electrodes in the C state and (**d**) the band structure after the Mott-to-band state conversion process.

From a Landau theory viewpoint, charge cannot couple directly to the order parameter *ψ* of the CDW, but can couple through Lifshitz-invariant spatial gradient terms in the free energy 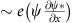, which are present at DWs. We propose that as the pulse of charges propagates through the sample, the energy of the system is lowered by charge carriers dynamically creating DWs. As the DWs are charged (with respect to the CDW background), they repel each other, creating a periodically textured stripe structure, an effect that is well known in correlated electron systems. The free energy *F* for such a system includes terms from the IC CDW formation and the DW repulsion, and has a number of minima, corresponding to states with different CDW and DW order (Fig. 4b) already in the absence of additional charge^20^. In the Supplementary Notes 4–5 and Supplementary Figs 6–7, we consider the situation in detail, showing that on charge injection the C state is unstable towards the formation of a periodic DW array, IC with the underlying lattice structure. The origin of this instability lies in the negative curvature of the inverse compressibility *k*=d^2^*F*/d*n*^2^<0 arising from the shape of *F*(*n*), which in turn is a consequence of the Coulomb repulsion between DWs. The periodicity of the DWs is directly related to the injected charge density *n*_i_=*n*_e_−*n*_h_: the resulting structure is commensurate if *n*_i_=0 and IC otherwise, with a wavevector *q*_H_=*n*_i_/*π* corresponding to the minimum in the free energy (Fig. 4b). The DW structure in the current-injection-induced H state is thus similar to the NC state, but with a periodicity that depends on *n*_i_. Inhomogeneity of charge injection will naturally lead to spatially inhomogeneous DW structures observed in experiments. Nevertheless, the state conversion from polarons to band states means that it is not only the DWs, but the entire state that is metallic.

Microscopically, a simple Mott–Hubbard picture^21^ is inadequate for a description of the DW formation^18^. However, if we also take into account the effect of long-range Coulomb repulsion and consider pulsed carrier propagation through a commensurate polaronic crystal (Fig. 4a), we can qualitatively understand the origin of the textured state also from the microscopic viewpoint. The carriers launched at the electrodes first lose their excess energy e*V*=*E*_e_−*E*_h_=1∼10 eV (Fig. 4c,d) within <1 ps. Taking into account scattering and energy loss to phonons of these energetic carriers gives an avalanche multiplication factor 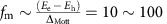. By the time the e and h reach the upper Hubbard band (UHB) and lower Hubbard band (LHB), respectively, the number of carriers injected by a 40-ps pulse is ∼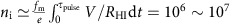. Occupancy of the Hubbard bands is unstable, so as they propagate in the applied electric field, the holes annihilate with the localized electrons at the centre of each polaron, leading to the creation of voids in the polaron lattice, accompanied by a dynamic conversion of polaronic (Mott–Hubbard) states to conducting band states as the wave travels through the sample (Fig. 4a). (STM studies suggest that electrons are not expected to show the same effect as holes^18^.) From the STM images (see Supplementary Note 6 for details), the number of holes *n*_h_ that can be accommodated in the DWs is very roughly between ∼1/4 and 1/3 of all the available polaron sites; thus, for our typical sample of volume 3 μm^3^ we estimate *n*_h_≈10^6^∼10^7^. This is consistent with the number of injected charges in the pulse *n*_i_. However, rather than forming a homogeneous patch of voids at the electrode, as a result of the Coulomb interaction, a textured state forms (Fig. 4a). The time for such an DW state to form after melting is known to be *τ*_form_∼1 ps (ref. 22), which is consistent with the observed speed of the switching process.

The observed independence on pulse length can thus be understood: the DWs are formed by the carriers in the leading edge of the pulse. Above a certain threshold, the entire sample is converted and further added charges have no additional effect; thus, the trailing edge of the pulse has no further effect. Importantly, the unusual exponential *I–V* curves below threshold described by equation (1) can be understood within this picture in terms of carrier tunnelling through barriers resulting from the transient DW structure. We show in the SM that the tunnelling rate within such a state leading to equation (1) is *R*∼exp(*V*/*V*_0_)^*β*^ where *β*∼1, where the barrier energy *V*_0_ is related to the C state order parameter, giving rise to its *T*-dependence shown in Fig. 3b.

The experiments introduce a paradigm of all-electronic switching between CDW states for data storage applications beyond current concepts^12^,^14^,^23^,^24^,^25^,^26^,^27^,^28^,^29^. Devices based on CDW state switching in 1T–TaS_2_ could be scaled and integrated into commonly used cross-bar configurations^30^. The demonstrated write speed ∼30 ps is externally limited, but is an order of magnitude faster than the previous record for non-volatile memory^14^,^25^. The intrinsic speed appears to be <1 ps. With *τ*_W_=30 ps, the expended energy per bit is estimated to be 
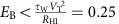
 pJ for a device with an area 2 × 2 μm^2^ (shown in Supplementary Fig. 8), which is extremely small, comparable with current magnetic random access memory (MRAM) devices^31^. As both *V*_T_ and *R*_HI_ scale linearly with distance between contacts (see Supplementary Fig. 9), *E*_B_ scales linearly with size along the contact direction and can be reduced by making the devices smaller. From a practical device viewpoint, it functions as a latch at low *T*. The reverse *M*–*I* transition is caused thermally, which requires that the device is heated momentarily above *T*_c2_—which can be done on a sub-nanosecond timescale with appropriate design^25^. Moreover, in practical devices bulk Erase is used where entire blocks are erased simultaneously; thus, *E* speed is not a limiting device operation speed. The device operating temperature appears to be limited by *T*_c2_ (Fig. 3a). It is known that this can be increased significantly by Se substitution: 1T–TaS_1.5_Se_0.5_ has a *T*_c2_>300 K (ref. 32), introducing the possibility of room temperature operation, whereas the relaxation time *τ*_R_ has been recently shown to be controllable by compressive strain^5^ tuning the data retention time of the device.

## Methods

### Sample preparation

1T–TaS_2_ crystals were grown by chemical transport method with iodine as a transport agent with the average lateral dimensions 1–5 mm and thicknesses ∼100 μm. Thin flakes were obtained from single crystal by exfoliating with sticky tape. The obtained 50- to 100-nm-thick flakes were deposited on sapphire substrate. Four gold electrodes were deposited on top of the flake by laser-direct lithography with initial 10-nm-thick Au/Pd layer.

### Electrical measurements

DC transport measurements were performed using standard four-point technique. All switching experiments were performed starting from the C Mott phase by applying single electrical pulse. The pulses in the range 100 ns–1 s were produced by a standard pulse generator. For 40-ps full width at half maximum pulse we used metal–semiconductor–metal current source triggered by a 35-fs laser pulse.

## Additional information

**How to cite this article**: Vaskivskyi, I. *et al*. Fast electronic resistance switching involving hidden charge density wave states. *Nat. Commun.* 7:11442 doi: 10.1038/ncomms11442 (2016).

## Supplementary Material

Supplementary Information Supplementary Figures 1-9, Supplementary Notes 1-6 and Supplementary References
