# Supplementary material for: Fast electronic resistance switching involving hidden charge density wave states
Source: Nat Commun. 2016 May 16;7:11442. doi: 10.1038/ncomms11442 (PMC4873615; doi:10.1038/ncomms11442)
Supplement: Supplementary Information — Supplementary Figures 1-9, Supplementary Notes 1-6 and Supplementary References [file ncomms11442-s1.pdf]

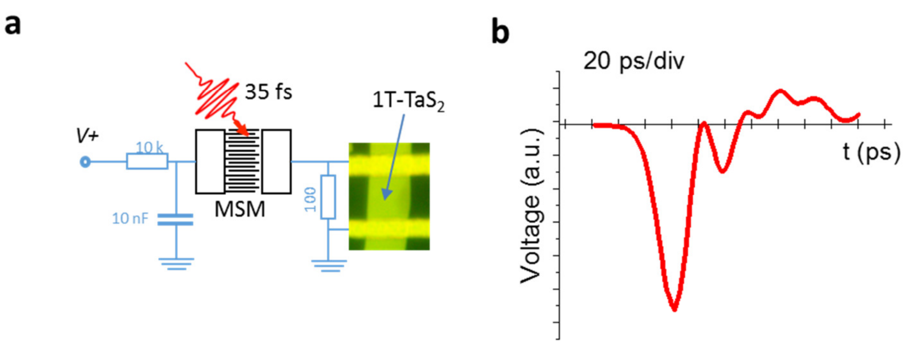

Supplementary Figure 1. Switching with 40 ps FWHM electrical pulse. a. The circuit diagram used for the measurement of the ultrashort pulse response. The distance between contacts is 2 μm. **b.** The measured electrical pulse risetime of the device, including cables in and out of the cryostat is 50±10 ps. b) the measured response of the circuit. The nominal risetime of the MSM source is 30 ps.

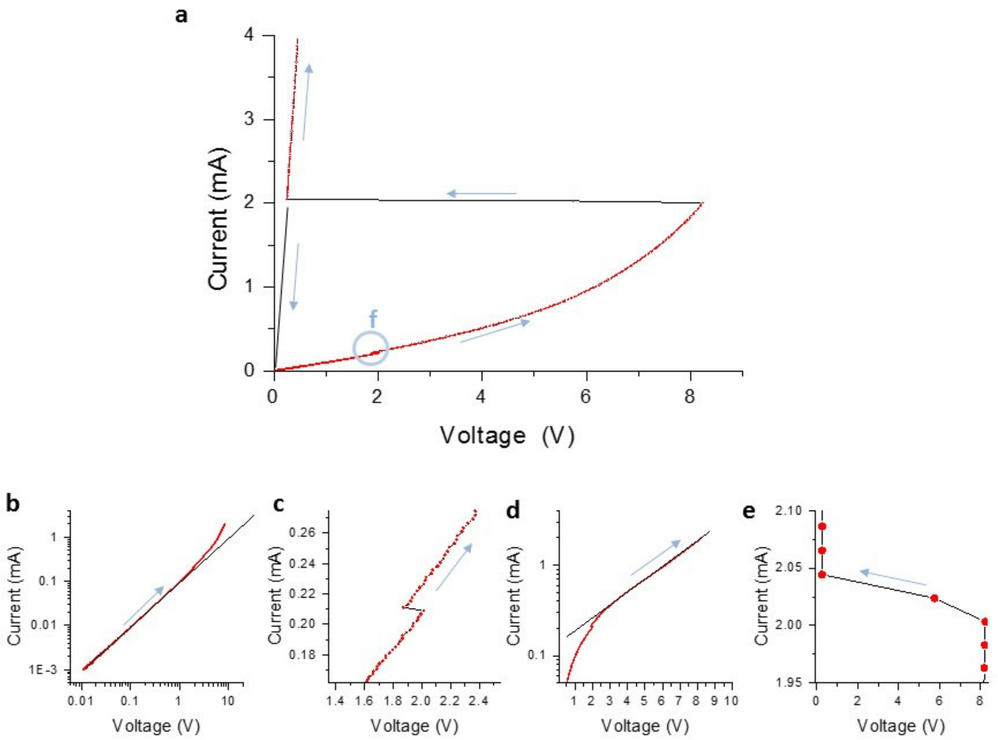

Supplementary Figure 2. Detailed features of the *I-V* curve. a. An *I-V* curve measured at 14 K in pulsed mode (τ_W=50 μs), where the current is incrementally increased with each pulse, and the voltage across the sample is measured during the pulse. The main features of the I-V curve are shown expanded: **b**, a linear *I-V* characteristic at low *V*. **c,** a sharp discontinuity is reproducibly visible in some samples. **d.** The *I-V* curve crosses over to an exponential one above ~ 3 V, as shown in the log-linear plot. **e.** The switching occurs in a narrow current interval, at V_T=2.05 mA for this sample.

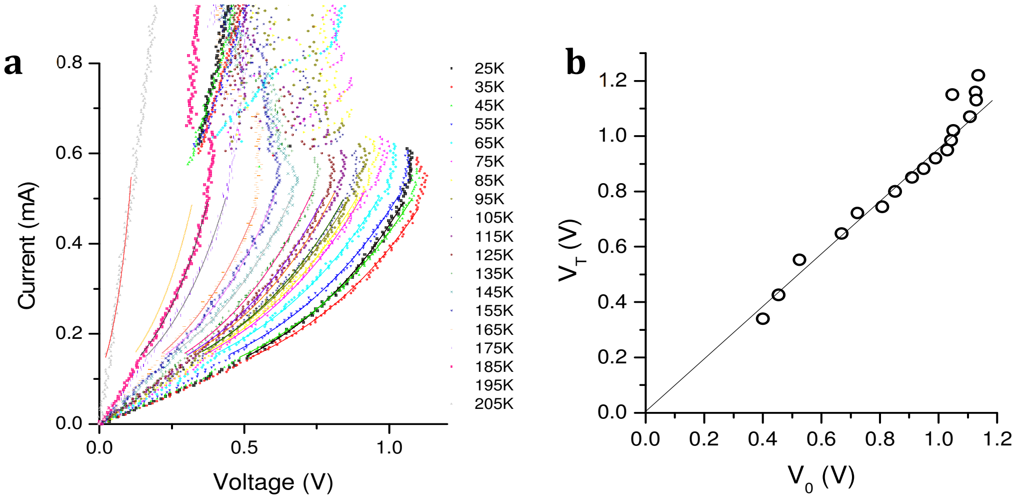

Supplementary Figure 3. Detailed fits to the data. a. Exponential fits to the data at different temperatures $25 < T < 205$ K are shown by solid lines. The parameters I_T and V_0 obtained from the fits are given in the main text (Fig. 3b). At intermediate temperatures we observe telegraph noise indicating thermally-activated switching between different resistance states. Note that the distance L between contacts is smaller than for the sample shown in Supplementary Figure 2, so V_T is smaller. **b**. The relation between V_0 and V_T obtained from the fits.

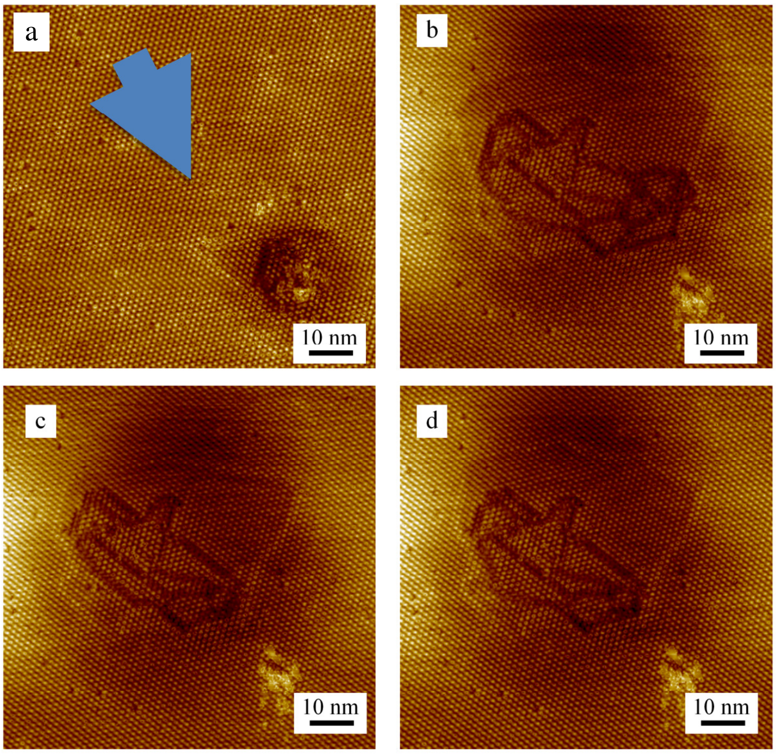

Supplementary Figure 4. STM images of the surface of 1T-TaS$_2$ a. before switching, **b-d.** after the switching. The arrow shows the nominal position of the tip. Images **b-d** show slow relaxation of the H state with time under continuous STM imaging.

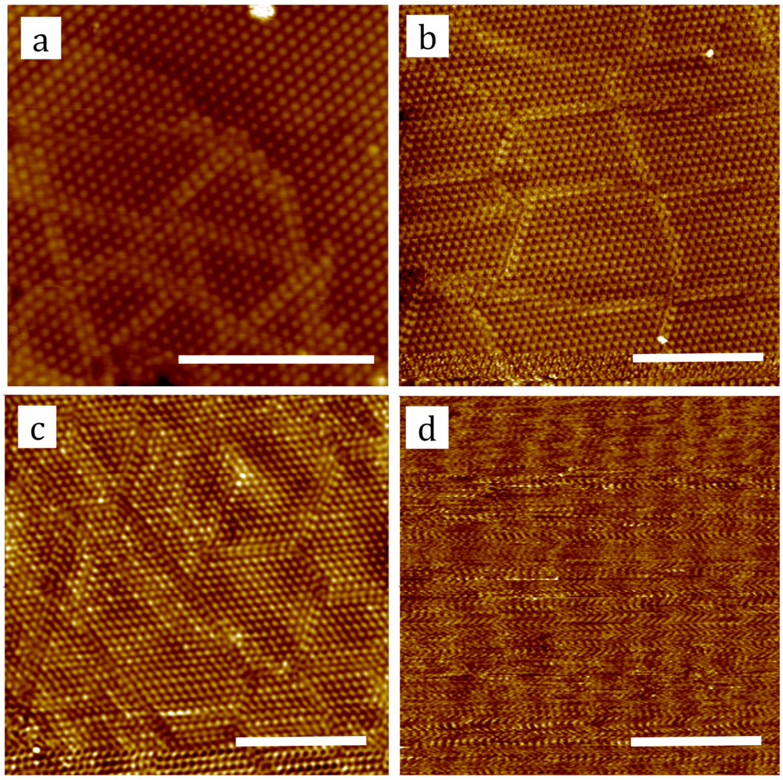

Supplementary Figure 5. STM images of the H structure in 1T-TaS_2 after switching at different temperatures: a. 4.5 K, **b.** 30 K, **c.** 50 K and **d.** 220 K. The scale bar signifies 20 nm. The image at 220 K shows no difference before and after the applied pulse, just the usual T phase which is present upon heating. All images were taken in constant current mode with a gap voltage 0.3 V and the current set point of 1 nA. Switching was achieved by voltage pulses with amplitude 7 V and a duration of 50 ms.

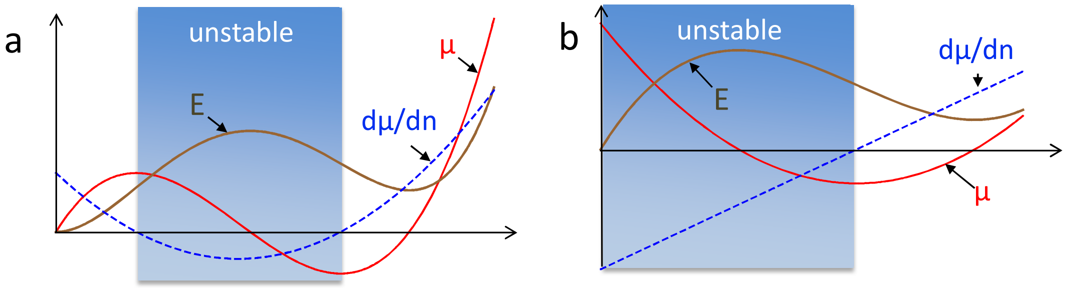

Supplementary Figure 6. Thermodynamic functions as a function of *n*. Plotted (in arbitrary units) for bi-stable systems near the 1st order phase transition: free energy E (brown), chemical potential μ (red), its derivative k (blue-dashed). **a.** The generic system. **b.** system with a C-NC transition. Note that the coordinate n assumes the opposite direction with respect to the wavevector q in plot in the main text (Fig. 4b).

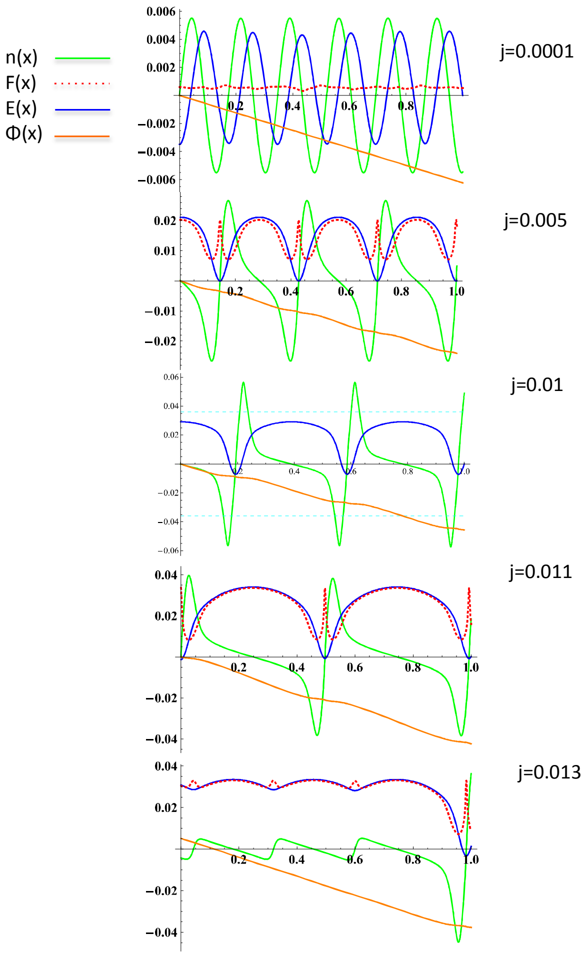

Supplementary Figure 7. **The numerical solution of the differential equation for the stripe phase.** Green curves: $n(x)$, blue curves: $E(x)$, red dotted curves: $G(x)$, orange curves: $\Phi(x)$. In these simulations, the current is increased progressively through values $j = 0.0001, 0.005, 0.01, 0.011, 0.013$.

The units are arbitrary, different for each plot to show all curves on a similar scale; only *E(x)* and *G(x)* are on the same scale. The magnitude of $n(x)$ progressively increases with *j*; for $j = 0.01$ and above, $n(x)$ crosses with levels (dotted lines) separating the domains of two equilibrium phases.

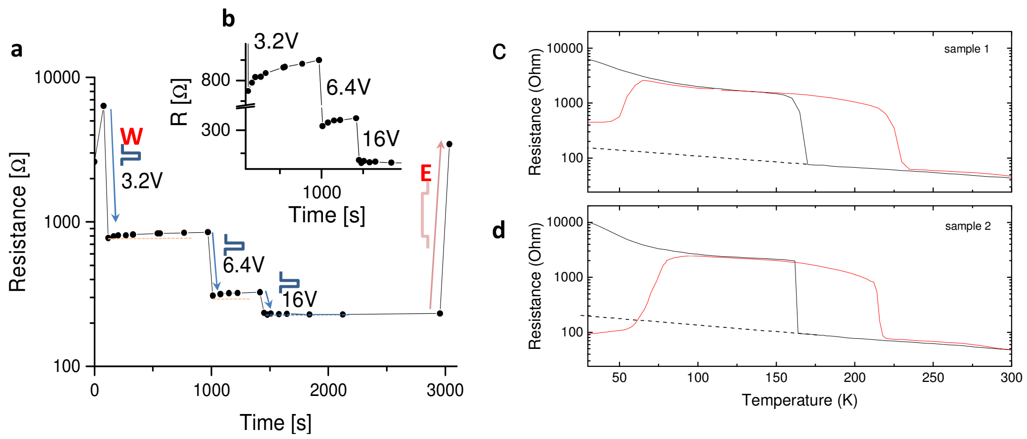

Supplementary Figure 8. The resistance of intermediate states below and above the extrapolated NC-state value. **a.** A resistance cascade through intermediate states obtained by gradually increasing the switching voltage near threshold (τ_W = 2 μs) for a device with a 3 μm gap between contacts at T = 4 K (note the logarithmic resistance scale). **b.** The relaxation of the intermediate states after 3.2 and 6.4 V pulses on a linear ordinate scale. The different relaxation properties of the observed states are there clearly evident. **c**. The temperature dependence of the resistance in the hidden state, with R above the extrapolated supercooled value corresponding to the NC phase. **d.** The temperature dependence of the resistance in the hidden state, with R below the extrapolated superculed value.

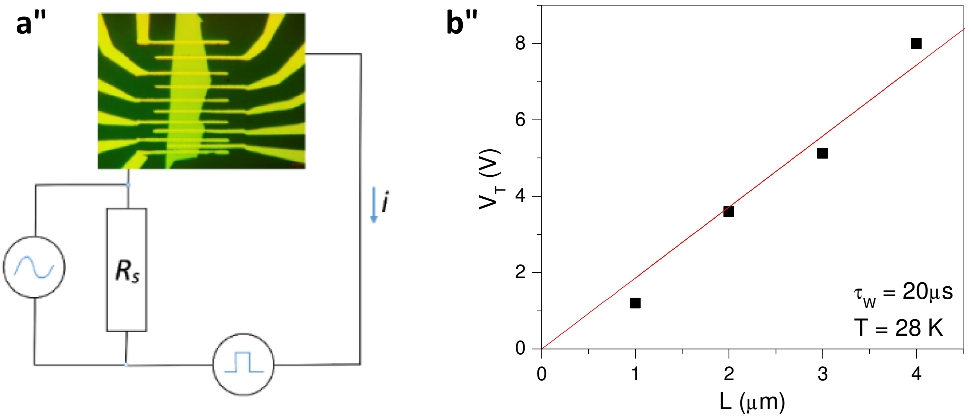

Supplementary Figure 9. The dependence of V_T on the distance between contacts *L*. **a.** photo of the device. A single flake with multiple contacts, with varying inter-contact distance was prepared. The resistance was always measured between adjacent contact electrodes. **b.** Dependence of V_T on distance between inner contacts L. The linear relation with an intercept at the origin indicates that no Schottky effects are present at the contacts.

Supplementary note 1. Device preparation and measurement methods.

Transport-grown single crystal samples between 20 and 120 nm thick, were deposited on sapphire substrates by exfoliating them with sticky tape and re-depositing onto the substrate. Multiple gold contacts were made using laser direct photolithography with a LPKF Protolaser LDI laser writer. A 5 nm Au/Pt intermediate layer beneath 100 nm thick gold electrodes was deposited by sputtering. Au is chosen because it is known to grow epitaxially on 1T-TaS_2, does not intercalate into it, does not give rise to any charge transfer and does not change the CDW of 1T-TaS_2.[1] Ohmic behaviour of the contacts on the C and NC state implies that there are no Schottky barriers at the contacts. The contact resistance was typically a few hundred ohms based on a comparison between 2 and 4 contact measurements.

The work functions of Au (W=5.1 – 5.47 eV), Pt (W=5.12-5.93 eV) and 1T-TaS_2 (W=5.2[2]-5.5 eV[3]) are well matched, although there is some uncertainty due to the surface characteristics of the sputtered Au and Pt. The measuring circuit and a typical sample with two contacts is shown in Supplementary Figure 1 for the case of an ultrafast MSM diode source, while Supplementary Figure 9a shows a sample with multiple contacts.

The resistance is measured either in 2 or 4 contact configuration, unless otherwise stated, with low currents < 1 μA to minimize Joule heating. The distance between contacts was typically between 1 and 8 μm. The switching threshold voltage is typically ~1V/μm. The switching was performed with electrical pulses using a Keithley measurement system (Keithley 6221 Current source + Keithley 2182A Nanovoltmeter) for write (W) pulse duration τ_W > 5 μs. For intermediate pulse durations 20 ns < τ_W < 5000 ns and pulses below 5 V we used a Stanford DG535 pulse generator, and an TI THS4211EVM amplifier or a standard pulse source, which behaves as a current source.

For pulses with V>5 V in Fig. 1 b of the main text, we used a SIGLENT SDG1025 function generator. (Note that the regulation circuit of this device produces a small ripple, which appears as an artefact in the form of steps seen in the V-t traces in Fig. 1b. of the main text). For pulses τ_W < 20 ns, we used a Hamamatsu MSM G4176-03 photodetector with a pulsed 35 fs laser source generating current pulses with a 30 ps rise/fall time (40 ps FWHM). The energy expended to switch from the C to the H state (energy per "bit") $E_B < \frac{V^2}{R}\tau_W \simeq 0.15$ pJ, where $V = 10$ V, $R = 20$ kΩ, $\tau_W = 30$ ps. The circuit is shown in Supplementary Figure 1. The I-V curves were measured in pulsed mode, using 50 μs W pulses in constant current mode and measuring the voltage across the sample during the second half of the pulse with the nanovoltmeter. The curves were measured by incrementally increasing the current.

With a pulsed constant current source, after the resistance switches to a lower value, the Joule heating I^2R drops and the switched state is not erased. With a constant voltage source, the Joule heating V^2/R increases after R drops, which may be sufficiently large to cause subsequent erase of the switched state, and the switching may appear to be volatile. The same applies to the use of a continuous constant voltage source, as used by Hollander et al [4].

The experiments were performed either in a Lake Shore 4-probe measuring station (to 4 K), or a closed cycle Oxford instruments cryostat (to 18 K). The ultrafast switching experiments were performed in a He-flow cryostat with semi-rigid 80 GHz bandwidth RF cable connections made directly to the sample.

Overall, more than 100 devices were prepared for these experiments, with excellent reproducibility, the main problem limiting successful operation being sample cracking at the first order NC-C transition on cooling.

We note that with a pulsed constant current source measurement, after the resistance switches to a lower value, the Joule heating I^2R drops and the switched state is not erased. With a constant voltage source, the Joule heating V^2/R increases after R drops, which may be sufficiently large to cause subsequent erase of the switched state, and the switching may appear to be volatile.

Supplementary Note 2. Detailed analysis of the I-V curve and fits to the data.

In Supplementary Figure 2 we analyze the features of an *I-V* curve in detail, measured at 14 K on a sample with the distance between contact electrodes of 8 μm. Each data point on the graph corresponds to a single voltage measurement taken during the applied current pulse as explained above in section 1.

Up to ~ 2 V, the *I-V* curve is close to linear (Supplementary Figure 2 b). At approximately 2 V, there is a small sharp discontinuity, where at I_{c1} = 0.21 mA the voltage drops by ~ 0.1 V (Supplementary Figure 2 c). This step is not seen at higher temperatures (see Supplementary Figure 2), and is sample dependent. We attribute it to switching to an intermediate state – for example caused as a slight polaron rearrangement - whose resistance is close to the C state. Thereafter (> 3 V) the current increases exponentially with V, up to V_T = 8.1 V, and can be described quite well by $I=I_T \exp(V/V_0)$ with V_0 = 7.1 V (Supplementary Figure 2 d). The exponential *I-V* characteristic with large V_T shown in Supplementary Figure 2 is not compatible with simple microscopic electronic transport models, and its functional form is very different than for a sliding CDWs[5-7]. At a threshold V_T ~ 8.1 V (which corresponds to a threshold current of I_T ~ 2 mA for this device), the voltage drops within a remarkably narrow current interval ΔI < 40 μA (Supplementary Figure 2 e).

The temperature-dependent measurements shown in Fig. 3 a of the main text and in Supplementary Figure 3 a were obtained by a tested protocol which ensures perfect reproducibility with temperature cycling and switching: First the sample was slowly cooled from 310 K to the temperature of measurement indicated in the plot. The measurements were performed in pulsed mode, increasing the set current with each pulse, and measuring the voltage during the second half of the pulse, as explained in Supplementary Note 1. After the highest value of current is reached at the end of each measurement, the sample was slowly heated in the cryostat to 310 K, whereupon the entire cycle was repeated at the next temperature.

Exponential fits to the data in the temperature range 25 – 205 K are shown in Supplementary Figure 3 a. The values of the parameter V_0 and I_T are shown in Fig. 1 c of the main text. The relation between V_0 and V_T is shown in Supplementary Figure 3 b. The slope is remarkably close to 1, with zero intercept.

Supplementary Note 3. Reaching intermediate states and their relaxation.

Incrementally increasing the voltage under constant voltage conditions, we find that several intermediate resistance states are reachable (Supplementary Figure 8 a). In the states reached following 3.2 and 6.4 V pulses, the resistance relaxes slightly in ~ 1000 s (insert Supplementary Figure 8 b). Remarkably, the lowest resistance state reached with V = 16 V shows no such relaxation, and the resistance remains constant after the W pulse. The different states thus clearly have different relaxation times. These data demonstrate that we are dealing with different H states which are distinct from a super-cooled NC state.

The temperature dependence of the resistivity in the H state after switching in different samples shows that the H state may have a resistance higher or lower than the

extrapolated value corresponding to a supercooled NC phase, indicating that different DW configurations are reached (Supplementary Figure 8 c,d). Apart from pulse characteristics, which resistance state is reached is somewhat sample and substrate dependent, as indicated by the strong dependence of the relaxation on the strain[8,9].

Supplementary Note 4. Tunneling of carriers within a charged sheet with domain walls.

The observed weak dependence of V_0 on T in Fig. 3 b of the main text implies the presence of a tunneling mechanism which departs from the usual Shockley diode equation for the current, $I = I_S(\exp(\frac{V}{nV_T}) - 1)$, where $V_T=kT/e$, I_S is the saturation current, T is the temperature, e is the elementary charge and k is Boltzmann's constant and typically n=1~2 is an ideality factor describing the device. Clearly such a thermally activated T-dependence is directly contradictory to our experiments, so we need to investigate tunneling mechanisms beyond the Shockley equation.

Generally, the tunneling rate through a barrier is exponential $R\sim \exp(-S)$, where for a local barrier $S(V){\sim}V^{1/2}$. For a wide barrier with a constant build-in electric field this becomes $S(V){\sim}V^{3/2}$. The square root dependence is usually observed in tunneling microscopy, while the 3/2 power law is well established in semiconductor physics, but neither are consistent with our data, where we see $S(V){\sim}V$.

This can be understood if we consider a simple layer of an uncompensated charge with density ρ. Then the Poisson equation $\Phi'' = -4\pi\rho$ gives a parabolic potential along the x direction which varies with distance as $\Phi(\mathrm{x}) = \Phi_\mathrm{t} - 2\pi\rho x^2$.

Rewriting, $\Phi(x) = V_\mathrm{T}(1 - (x/l)^2)$ where $l^2 = \Phi_\mathrm{t}/2\pi\rho$ is the effective width of the barrier. In such a case, the exponent S in the transmission rate becomes linear in V:

$$S = \int \left[\frac{2m}{\hbar^2}(V - \Phi(x)\right]^{1/2} dx \sim \sqrt{\frac{2m}{\hbar^2 V_\mathrm{t}}}\, l(V_\mathrm{t} - V) \qquad (1)$$

where m is the effective mass of the electron, $V_0 = \sqrt{\frac{\hbar^2 V_\mathrm{t}}{ml^2}}$, and $V_\mathrm{t} = \frac{ml^2V_0^2}{\hbar^2}$. With $V_\mathrm{t} \sim V_0$ of order of 1 eV, and m=$m_\mathrm{e,}$ the barrier width, $l \sim 3$ Å is of the order of the polaron radius. For $V > V_\mathrm{t}$ there is no more reflection from the barrier, giving rise to a high

conductivity. The electrons spill over the entire sample transforming it into the H state which is stable even after the voltage is released.

Usually uncompensated charges do not occur, since conductors are everywhere close to local electro-neutrality. But stripes or domain walls may introduce such charges, stabilized by the energy gain arising from the formation of the stripe phase. An example of modeling for an equilibrium structures in a 2D charged system of polarons, can be found in [10] and references therein.

Microscopically, the barrier may be related to the tunneling of electrons through charged stripes and between polaron clusters. Its temperature dependence is related to the temperature dependence of the sheet charge density in the C state $\rho(T)$, which is in turn loosely related to the order parameter of the C phase $\Delta(T)$. Thus, at low T, all the sites are occupied and the charge density is maximum. As $T \rightarrow T_{c2}$ when the material becomes conducting in the NC phase, the excess charge density $\rho(T) \rightarrow 0$.
Although this simple picture cannot explain the T-independent I_T, taken as an empirical fact it leads to the T-dependent decrease of V_T simply through the dependence of resistance on T.

Supplementary Note 5. Instability towards stripe formation in 1T-TaS$_2$ after charge injection.

The starting point for the phenomenological modelling is the model of Nakanishi and Shiba[11-13], which has the advantage that it has been thoroughly tested by structural and thermodynamic studies, and its parameters have been carefully ascertained. We proceed by limiting ourselves to the customary one-dimensional model (for simplicity), assuming that the in-plane ordering occurs identically along the three directions according to the underlying hexagonal symmetry.

First, let us start with a brief description of the behavior of a generic bistable system in between two contacts. Commonly, a potential applied to a sample is expected to concentrate the local charge density at the contacts so that charge is screened completely or partly (if the current keeps flowing between the contacts).

In our special situation the charge penetrates into a bistable medium in which the competition of phases is controlled by the particle density n. Here n is meant to describe the hole density n_{H} in the main text, but in general refers to the defect density, where these can be either holes or extra electrons. The free energy F of this state in equilibrium is shown in Fig. 4 b of the main text, and in more detail in Supplementary Figure 6 a (brown curve). To aid further discussion, we show the chemical potential $\mu = \frac{dE}{dn} = F'$ (red curve) and the inverse compressibility $k = \frac{d\mu}{dn} = \frac{d^2F}{dn^2}$.

Recall that $k > 0$ is the condition of the thermodynamic stability [14]. We see in Supplementary Figure 6 a that conventionally both phases are stable ($k > 0$, where $E(n)$ is convex); only the transient region around the barrier between zeros of k is unstable. Above a supercritical concentration (which can arise near the contact junction) a layer will appear when the system sits in the metastable high n state, keeping the low n ground state in the bulk. As far as we are aware, such a phase transformation at the contact boundary has not yet been considered, except in the recent context of the electrostatic doping when very high fields are involved[15].

The situation is more unusual if we consider, as it seems to be the case here, that the charges are not in band states, but form commensurability defects in the polaronic lattice of the C state.

The energy $E(n)$ dependence on concentration n of defects again has a double-well structure with the minimum at $n = 0$ and another minimum at a finite n, see Supplementary Figure 6 b. But now there is a principle difference: unlike the natural GL

case of Supplementary Figure 6 a, the energy $E(n)$ at small n has a cusp, it starts as $W{\sim}n$ and then decreases. The curve $W(n)$ is now concave around the low n phase, and compressibility is now negative $k = d\mu/dn < 0$ for all n up to the inflection point of $W(n)$ which lies between the barrier and the high n minimum (the drawn vertical line in Supplementary Figure 6 b). Particularly for a charged system, this violation of the stability criterion means that the screening length square becomes negative:

$$\left(\frac{1}{l_{\mathrm{scr}}}\right)^2 = \frac{4\pi e^2}{\Omega}\left(\frac{dn}{d\mu}\right) < 0 \qquad (2)$$

where Ω is the unid volume per charge. When the above expression is negative, l_{scr} becomes imaginary. That leads to oscillations instead of the conventional exponential decay with distance, resulting in phase separation and stripe formation. The implication is that the system is approaching a 1st order C-IC transition which is locally unstable, but is protected by charge conservation and further stabilized by the boundary conditions. When charges are injected by the current, the instability is realized and a stripe pattern is formed. This can describe the observed nearly T-independent behavior for $I < I_{\mathrm{T}}$. Since the stripe period (except when approaching I_{T}) is an intrinsic property, the total voltage drop will be the sum of drops across each stripe which is proportional to L, as shown in Supplementary Figure 9 b. The tunneling across the charged stripe, which provides a parabolic barrier, will give an exponential law with $\beta = 1$ as discussed above.

These general qualitative statements can be verified and illustrated by means of a simple model. We shall work in a way which is compatible with the previous modeling of the homogeneous time evolution towards the H phase in optically excited experiments [16]. Still there are important differences between optical and current experiments, which are not completely resolved. The principle difference is that in optical switching the density of both photoexcited electrons and holes is initially very high, and their temperature is also high. This allowed the system to easily find the minimum in free energy corresponding to the H phase. This is not the case with current

injection. Rather, with strongly inhomogeneous, spatially undulating distributions of charges and fields the highly nonlinear boundary value problem becomes more difficult to solve.

To calculate the spatial density distribution under the influence of a current, we shall simplify the function $F(n)$ as

$$F(n) = E_{\mathrm{d}}(1.05|n| - 20n^2 + 100|n|^3 \tag{3}$$

where E_{d} is close to the defect formation energy (our earlier estimation was $E_{\mathrm{d}}\approx 0.1$ eV [16]). The plot of $\boldsymbol{F(n)}$ for small n (see Supplementary Figure 6 a) closely reproduces the free energy previously obtained for the homogeneous system[16], with a minimum of $W(n)$ at $n \approx 0.1$. The chemical potential and the inverse compressibility of defects become:

$$\boldsymbol{\mu(n) = dF/dn = E_d\ (1.05\ sgn(n) - 40n + 300n^2 sgn(n))} \tag{4}$$

$$\mathbf{k(n) = d\mu/dn} = \boldsymbol{E_d}\ (\mathbf{-40 + 600|n|}) \tag{5}$$

These functions are plotted in Supplementary Figure 7 b. The total electro-chemical potential is:

$$\mu_{\mathrm{tot}}(x) = \mu\big(n(x)\big) + e\Phi(x), \qquad \varepsilon_0\Phi'' = -4\pi e n_3 \tag{6}$$

Here the electrostatic potential Φ satisfies Poisson's eq. where e is the elementary charge, ε_0 is the host dielectric constant and $n_3 = n/\Omega$ is the concentration of particles per unit volume ($\Omega = ds, s = 13s_0$ is the unit area per polaron in the C phase and d is the interplane distance; recall that the dimensionless n is a relative concentration of defects).

The gradient of μ_{tot} determines the current density $j(x)$ which is constant $j(x) = j$ in the stationary regime:

$$j(x) = -\sigma(n(x))\partial_x \mu_{\text{tot}}(x) = j = const \quad (7)$$

For the conductivity $\backslash signa(n)$ we chose the regime of constant mobility b: $\sigma(n) = bn$ where $b = const$ (numerically a finite residual value is kept for σ when n passes through zero).

The characteristic length generalizing the qualitative notion of the imaginary screening length l_{scr}, appears from these equations as $l^{-2} = 4\pi e^2/(40\varepsilon_0 dsE_{\text{d}})$ which yields $|l| = 0.4\sqrt{s\varepsilon_0}$ in units of distance between polarons. The in-plane static dielectric constant ε_0 (which is not known experimentally but is expected to be high, $\varepsilon_0 \sim 10^2$, for 1T-TaS_2 with its highly polarizable structure) can enhance the value of l by an order of magnitude above the basic period- the bare distance between polarons $\sqrt{s}$.

The system of Supplementary equations (5,6,7) has been solved numerically for several values of j with boundary conditions: $n(0) = 0$ at the sample center, defined as $x = 0$ and $E(1) = 0$ at the sample boundary (the contact) at $x = 1$. (The units are in μm.) The parameter $I = 0.03$ was enhanced for demonstration purposes (to reduce the number of oscillations). The solution yields a periodic function oscillating in space, instead of the conventional exponential decay with distance from the electrodes. The main reason for this is the non-linear behavior of $\mu(n)$ with the unconventional curvature $\mathrm{d}^2F(n)/d\mathrm{n}^2 < 0$.

The calculated patterns of $n(x)$, $E(x)$, $G(x) = -\partial_x \mu_{\text{tot}}$ and $\Phi(x)$ for different values of current j are presented in plots of Supplementary Figure 7. They show undulations between hole-doped and electron-doped domains, with $n > 0$ and $n < 0$ respectively. The patterns evolve from a nearly sinusoidal shape to sharp domains whose number decreases progressively with increasing j, until the last strong one remains near the contact at $x = 1$ ($x = 0$ is the middle of the sample) while the bulk is converted to a highly conducting state with smooth weak undulations. At low j, the period does not

vary much with j and stays close to the value $\sim 2\pi l$ intrinsic to the system. The amplitude of $n(x)$ is relatively small – the system is always close to the C phase where $n = 0$. With increasing j, the amplitude extends to, and even beyond the barrier height at $n_{\mathrm{B}} = \pm 0.036$ (indicated by the dotted horizontal line in Supplementary Figure 7 shown in the plot with $j = 0.01$) separating the NC phase at $n_{\mathrm{NC}} \approx 0.1$. This nonlinear regime shows abrupt steps reducing the period of undulations – the last *N=3* remnant oscillations are shown in Supplementary Figure 7 e, until the undulations disappear completely, being washed out by the current. The value of *N* is subject to boundary conditions imposed by the contacts, and changes discretely, which is demonstrated by our modelling. The occasional jumps observed in the I-V curve are thus understood, as well as the steps in the relaxation.

Until now we did not consider the normal carriers, assuming that at modest concentration their presence will be reduced to modifications of numerical parameters of our model, similar to thermal effects. For example, the mobility b of defects may not reflect their actual motion but can be promoted by normal carriers which (re)condense to/from defects depending on the game of relative electro-chemical potentials. The normal carriers can appear explicitly due to an asymmetry of properties of voids and interstitials when the charge compensation between opposite domains cannot be maintained by defects alone. Also with rising of domains length with increasing current, their mutual neutralization is not sufficient and the demand for the local charge compensation will give rise to an appreciable concentration of band carriers within each domain. The conduction by the carriers requires tunneling through a striped sheet of charges for which the rate have been shown in section 6 to be exponential $\Gamma \sim \exp \frac{V}{V_0}$, leading to $I/I_0 = \exp(V/V_0)$, as observed.

Our modeling so far has not yet demonstrated persistence of the resulting mono-domain structure after the current is turned off. To do this, time dependent equations

with extended degrees of freedom (normal carriers taken explicitly, and a variable amplitude of the crystalline order parameter), need to be solved which is beyond the scope of this publication. But it is already apparent that the obtained protrusions of *n(x)* beyond the interphase barrier (crossing with dashed lines as shown for j=0.01 in Supplementary Figure 7) will locally nucleate the new phase. It remains to be understood if the nuclei will coalesce to give a macroscopic domain or the phase will keep the undulating structure. In the latter case, to conform with the lattice symmetry of 1T-TaS_2, the implied 1D stripe order would be three directional, at 60 degrees to each other, leading to a honeycomb pattern as schematically indicated in Fig. 4 a of the main text.

In summary, the presented calculation clearly demonstrates the propensity of the system towards the formation of a textured stripe-like conducting state induced by the presence of a current, and indicates the origins of its metastability.

Supplementary Note 6. The domain walls in the H state confirmed by scanning tunneling microscopy (STM)

To reveal the microscopic structure of the H state we mapped the surface of the material in the C and H state by means of STM (Omicron LT nanoprobe). The samples were first cleaved in-situ under the UHV conditions. The imaging of the samples at T=4.5 K was then performed by a W or Pt/Ir tunneling tip with a tunneling current I_t=50 pA and a gap voltage of V_{gap}=-0.65 V. To switch to the H state, the tip was placed in tunneling contact with the sample and a 1 ms voltage pulse (5-10 V) of similar magnitude as in switching experiments described in the main text was applied. The geometry is similar to the lithographic contacts, in the sense that current is injected from the top surface, but the surface area is smaller. Afterwards, the STM image was obtained at the same operating conditions as before the switching pulse.

The typical image of the sample in the C state (before switching) is shown in Supplementary Figure 4a. It reveals the periodic hexagonal CCDW ordered texture. Applying the voltage pulse causes the part of the sample beneath the tip to switch to the H state. The new state is characterized by a modified patterned structure (Supplementary Figure 4b), which consists of relatively small domains (~10x10 nm) with a CDW modulation similar to the C state. The phase of the CDW changes abruptly between the domains, which causes the formation of domain walls. We noticed a few different types of walls depending on their directions, consistent with [17,18]. While no relaxation of the structure was observed without external influence, under continuous STM imaging the area of patterned phase decreases with time. The relaxation is accompanied by the motion of the domain walls towards the center of the cluster and their gradual disappearance (Supplementary Figure 4b-d). The relaxation occurs in single-polaron steps, consistent with the hypothesis presented by Vaskivskyi et al[8].

The whole surrounding area around the H state is darker due to the modified electronic structure of the material. Scanning tunneling spectroscopy reveals closure of the Mott gap in the H state not only inside the domain walls but also for the CDW inside the domains [17,18], confirming the metallic behavior we observe in the transport measurements presented in the main text.

Warming the sample in the H state results in a gradual thawing of the domain structure in the H state. STM images at 4.5 K, 30 K, 50 K and 220 K respectively are shown in Supplementary Figure 5. The thawing of domains at high temperatures is consistent with the behavior of the V-I curves at different temperatures shown in Fig. 3a of the main text. The 220 K image shows the formation of the striped T phase, previously observed by Thomson et al.[19].

Supplementary references

[1] Shimada, T. Fabrication, Structure and Physical Properties of Van Der Waals Interfaces. Ch. 7, PhD. thesis, Univ. of Tokyo (1993).

[2] Shimada, T., Ohuchi, F. S. & Parkinson, B. A. Work Function and Photothreshold of Layered Metal Dichalcogenides. *Jpn. J. Appl. Phys.* **33,** 2696–2698 (1994).

[3] Perfetti L. *et al.* Femtosecond dynamics of electronic states in the Mott insulator 1T-TaS2 by time resolved photoelectron spectroscopy. *New J. Phys.* **10**, 053019 (2008).

[4] Hollander, M. J. *et al.* Electrically driven reversible insulator-metal phase transition in 1T-TaS2. *Nano Lett* **15**, 1861-1866 (2015).

[5] Bardeen, J. Classical versus quantum models of charge-density-wave depinning in quasi-one-dimensional metals. *Phys. Rev. B* **39**, 3528-3532 (1989).

[6] Gruner, G. *Density Waves in Solids* (Addison-Wesley, 1994).

[7] Monceau, P. Electronic crystals: an experimental overview. *Adv Phys* **61**, 325-581 (2012).

[8] Vaskivskyi, I. *et al.* Controlling the metal-to-insulator relaxation of the metastable hidden quantum state in 1T-TaS2. *Science Advances* **1**, e1500168 (2015).

[9] Svetin, D. *et al.* Transitions between photoinduced macroscopic quantum states in 1T-TaS2 controlled by substrate strain. Appl. Phys. Express **7**, 103201 (2014).

[10] Miranda, J. and Kabanov, V. V. Coulomb frustrated first order phase transition and stripes. *Physica C: Superconductivity* **468**, 358-361 (2008).

[11] Nakanishi, K. and Shiba, H. Domain-like Incommensurate Charge-Density-Wave States and the First-Order Incommensurate-Commensurate Transitions in Layered Tantalum Dichalcogenides. I. 1T-Polytype. *J. Phys. Soc. Japan* **43**, 1839-1847 (1977).

[12] Nakanishi, K., Takatera, H., and Yamada, Y., Shiba, H. The Nearly Commensurate Phase and Effect of Harmonics on the Successive Phase Transition in 1T-TaS2. *J. Phys. Soc. Japan* **43**, 1509-1517 (1977).

[13] Nakanishi, K. and Shiba, H. Theory of Three-Dimensional Orderings of Charge-Density Waves in 1T-TaX2 (X: S, Se). *J. Phys. Soc. Japan* **53,** 1103–1113 (1984).

[14] Landau, L. D. and Lifshits, Y. M. *Landau: Statisticheskaya Fizika [Statistical Physics]* (Elsevier, 2013).

[15] Ueno, K. *et al.* Electric-field-induced superconductivity in an insulator. Nature Materials **7**, 855-858 (2008).

[16] Stojchevska, L. *et al.* Ultrafast switching to a stable hidden quantum state in an electronic crystal. *Science* **344,** 177–180 (2014).

[17] Cho, D. *et al.* Nanoscale manipulation of the Mott insulating state coupled to charge order in 1T-TaS2. *Nat. Commun.* **7,** 10453 (2016).

[18] Ma, L. *et al.* A metallic mosaic phase and the origin of Mott insulating state in 1T-TaS2. *Preprint at http://arxiv.org/abs/1507.01312* (2015).

[19] Thomson, R., Burk, B., Zettl, A. & Clarke, J. Scanning tunneling microscopy of the charge-density-wave structure in 1T-TaS2. *Phys. Rev. B* **49,** 16899–16928 (1994).
